# Impact of ^68^Ga-FAPI PET/CT on Staging and Oncologic Management in a Cohort of 226 Patients with Various Cancers

**DOI:** 10.2967/jnumed.123.266046

**Published:** 2023-11

**Authors:** Stefan A. Koerber, Manuel Röhrich, Leon Walkenbach, Jakob Liermann, Peter L. Choyke, Christoph Fink, Cathrin Schroeter, Anna-Maria Spektor, Klaus Herfarth, Thomas Walle, Jeremie Calais, Hans-Ulrich Kauczor, Dirk Jaeger, Juergen Debus, Uwe Haberkorn, Frederik L. Giesel

**Affiliations:** 1 Department of Radiation Oncology, Heidelberg University Hospital, Heidelberg, Germany;; 2 National Center of Radiation Oncology, Heidelberg Institute of Radiation Oncology, Heidelberg, Germany;; 3 Clinical Cooperation Unit Radiation Oncology, German Cancer Research Center, Heidelberg, Germany;; 4 Department of Radiation Oncology, Barmherzige Brueder Hospital Regensburg, Regensburg, Germany;; 5 Department of Nuclear Medicine, Heidelberg University Hospital, Heidelberg, Germany;; 6 Department of Nuclear Medicine, Mainz University Hospital, Mainz, Germany;; 7 Molecular Imaging Program, Center for Cancer Research, National Cancer Institute, National Institutes of Health, Bethesda, Maryland;; 8 Department of Medical Oncology, National Center for Tumor Diseases, Heidelberg University Hospital, Heidelberg, Germany;; 9 Clinical Cooperation Unit Virotherapy, German Cancer Research Center, Heidelberg, Germany;; 10 German Cancer Consortium, Heidelberg, Germany;; 11 Ahmanson Translational Theranostics Division, Department of Molecular and Medical Pharmacology, David Geffen School of Medicine at UCLA, Los Angeles, California;; 12 Department of Diagnostic and Interventional Radiology, Heidelberg University Hospital, Heidelberg, Germany;; 13 Clinical Cooperation Unit Nuclear Medicine, German Cancer Research Center, Heidelberg, Germany;; 14 Department of Nuclear Medicine, Medical Faculty, Heinrich-Heine University, University Hospital Düsseldorf, Düsseldorf, Germany; and; 15 Institute for Radiation Sciences, Osaka University, Osaka, Japan

**Keywords:** FAPI, PET/CT, management, staging, radiation therapy

## Abstract

Since the development of fibroblast activation protein–targeted radiopharmaceuticals, ^68^Ga-fibroblast activation protein inhibitor (FAPI) PET/CT has been found to be suitable for detecting primary and metastatic lesions in many types of tumors. However, there is currently a lack of reliable data regarding the clinical impact of this family of probes. To address this gap, the present study aimed to analyze the clinical impact of ^68^Ga-FAPI PET/CT by examining a large cohort of patients with various tumors. **Methods:** In total, 226 patients (137 male and 89 female) were included in this retrospective analysis. Pancreatic cancer and head and neck cancers were the most common tumor types in this cohort. TNM stage and oncologic management were initially determined with gold standard imaging, and these results were compared with ^68^Ga-FAPI PET/CT. Changes were classified as major and minor. **Results:** For 42% of all patients, TNM stage was changed by ^68^Ga-FAPI PET/CT results. Most of these changes resulted in upstaging. A change in clinical management occurred in 117 of 226 patients. Although a major change in management occurred in only 12% of patients, there was a significant improvement in the ability to accurately plan radiation therapy. In general, the highest clinical impact of ^68^Ga-FAPI PET/CT imaging was found in patients with lung cancer, pancreatic cancer, and head and neck tumors. **Conclusion:** ^68^Ga-FAPI PET/CT is a promising imaging probe that has a significant impact on TNM stage and clinical management. ^68^Ga-FAPI PET/CT promises to be a crucial new technology that will improve on conventional radiologic imaging methods such as contrast-enhanced CT and contrast-enhanced MRI typically acquired for cancer staging.

## Visual Abstract

**Figure:**
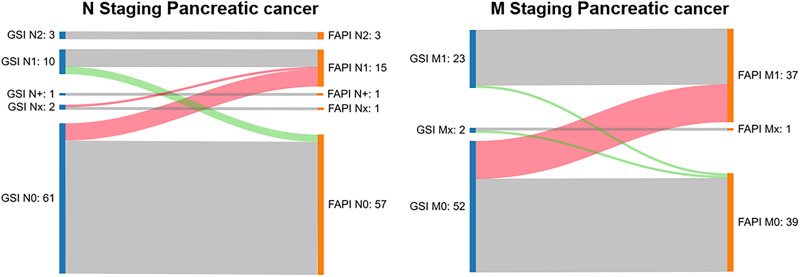

Individualized treatment approaches and personalized medicine play a crucial role in modern oncology. Accurate staging and restaging are essential for making informed clinical decisions in oncology. Over 40 y ago, ^18^F-FDG PET/CT emerged as an integral imaging probe for various tumors, such as lung cancer. In 1999, Nestle et al. reported a reduction in the size of radiotherapy portals based on ^18^F-FDG PET/CT information in a small retrospective cohort of lung cancer patients (1). Since then, ^18^F-FDG PET–based radiotherapy planning has demonstrated improved treatment efficacy, reduced observer variation, and improved local control without increasing toxicity rates (2–4). Although ^18^F-FDG has sufficient sensitivity and specificity, it has some well-known limitations. Physiologic ^18^F-FDG uptake in organs such as the brain or pharyngeal lymphoid tissue can hinder tumor detection in these anatomic regions (5). Additionally, false-positive uptake can be observed in activated brown fat and inflammation (6). Moreover, certain tumor types with low glucose transporter or hexokinase activity are not suitable for ^18^F-FDG PET/CT (7). Therefore, there is a need for novel tracers that can be widely used for patients with malignant tumors.

Considering that fibroblast activation protein (FAP) is highly expressed by stromal fibroblasts in more than 90% of epithelial cancers, radiolabeled FAP inhibitor (FAPI) tracers have shown promising diagnostic performance for oncologic imaging (8). Initial clinical results have demonstrated high uptake and image contrast in several tumor types, detecting many more lesions than conventional imaging (9*,*^10^). Numerous trials have confirmed the efficacy of ^68^Ga-FAPI PET/CT as an efficient imaging probe and have suggested its superiority over ^18^F-FDG for certain tumors (7*,*^11^*,*^12^). However, the impact of ^68^Ga-FAPI PET/CT on clinical practice remains unclear, with only a few small trials assessing the impact on staging and oncologic management (13*,*^14^). Here, we evaluated the role of ^68^Ga-FAPI PET/CT on TNM staging and oncologic management in a large retrospective patient cohort across multiple types of solid tumors.

## MATERIALS AND METHODS

### Data Collection

**FIGURE 1.**
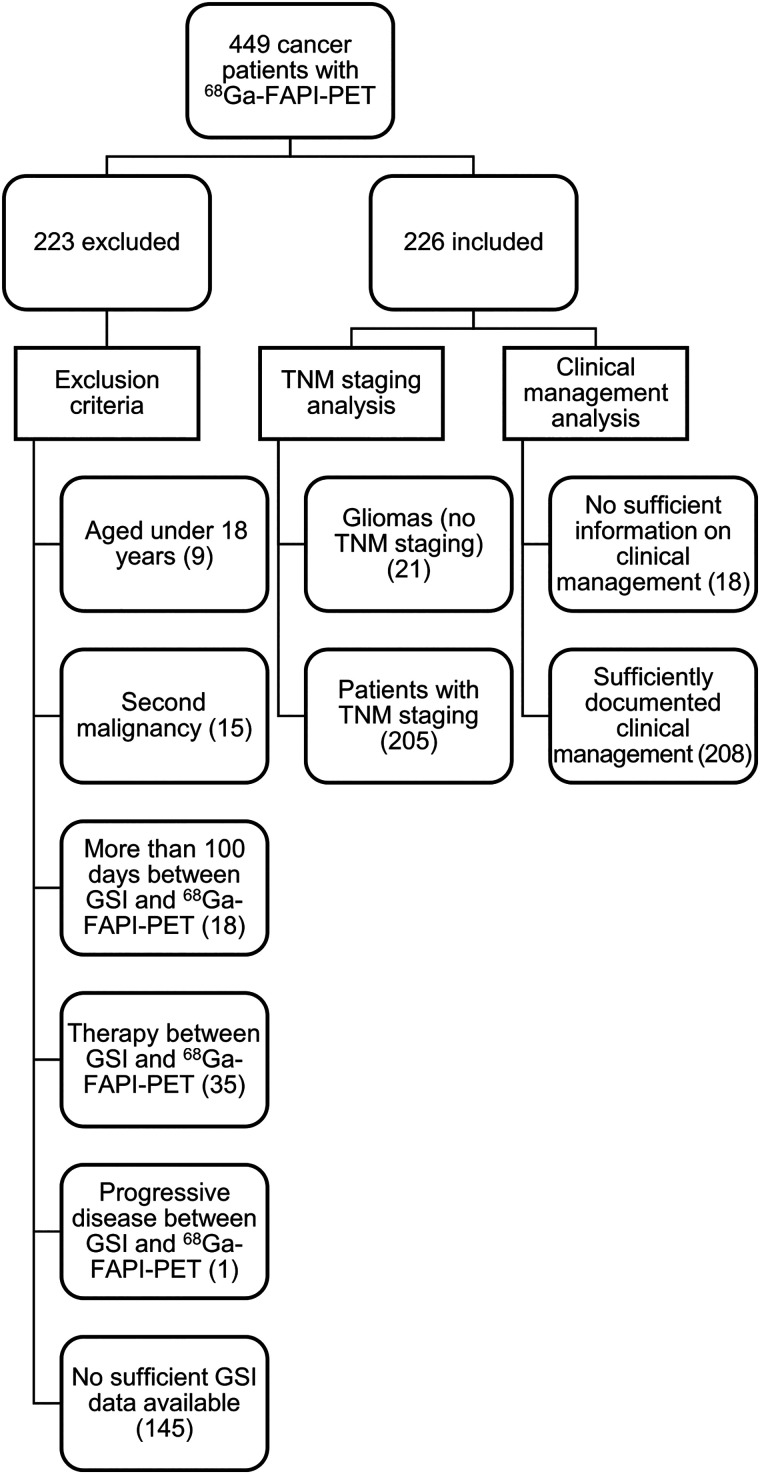
Flowchart displaying distribution of exclusion criteria among 449 cancer patients who underwent ^68^Ga-FAPI PET/CT at University Hospital Heidelberg between June 2017 and February 2022 (left side) and fractions of included patients who could be analyzed with regard to TNM staging and clinical management.

Between June 2017 and February 2022, 449 patients with various cancers were referred for ^68^Ga-FAPI PET/CT imaging. All patients underwent conventional gold standard imaging (GSI). ^68^Ga-FAPI PET was also performed to address issues such as inconclusive findings on other imaging modalities or to assist in radiotherapy planning. Of the 449 patients initially referred, 226 were selected for this retrospective analysis on the basis of the following inclusion criteria: an age of 18 y or older, adequate GSI data available, no secondary malignancy within 5 y, and an interval of less than 100 d between GSI and ^68^Ga-FAPI PET, with no intervening therapy and no evidence of progression between GSI and ^68^Ga-FAPI PET (Fig. 1). The local institutional review board approved this retrospective analysis (study S-430/2022). A subgroup of patients analyzed here were included in previous projects with small and midsize patient cohorts, in which we evaluated the impact of ^68^Ga-FAPI PET/CT on the staging and clinical management of pancreatic ductal adenocarcinomas (15) and adenoid cystic carcinomas (16) but not the impact on staging or clinical management of ^68^Ga-FAPI PET/CT for various cancer diseases (9*,*^17^–^21^).

### ^68^Ga-FAPI PET/CT Imaging

Four tracer variants of ^68^Ga-FAPI were used in this study: ^68^Ga-FAPI-02 (21 patients), ^68^Ga-FAPI-04 (63 patients), ^68^Ga-FAPI-46 (101 patients), and ^68^Ga-FAPI-74 (41 patients). All tracers were synthesized and labeled as previously described (22–24). A Siemens Biograph mCT Flow scanner was used for PET imaging, according to previously published protocols (10). Briefly, a low-dose CT scan with or without contrast medium was first obtained, followed by a 3-dimensional PET acquisition (matrix, 200 × 200). After image reconstruction, emission data were corrected for attenuation, scatter, and decay. All PET scans were acquired 60 min after administration of 200 ± 50 MBq of ^68^Ga-labeled FAPI tracers.

### TNM Staging Based on ^68^Ga-FAPI PET Compared with GSI

Staging guidelines were based on the eighth edition of the TNM classification of malignant tumors of the Union for International Cancer Control based on GSI and ^68^Ga-FAPI PET/CT findings by 1 board-certified radiologist, 1 board-certified radiation oncologist, and 2 board-certified nuclear medicine physicians in consensus. Staging was based on reviewing clinical imaging records, but if the written record was inadequate, images were reviewed by three of the authors.

**FIGURE 2.**
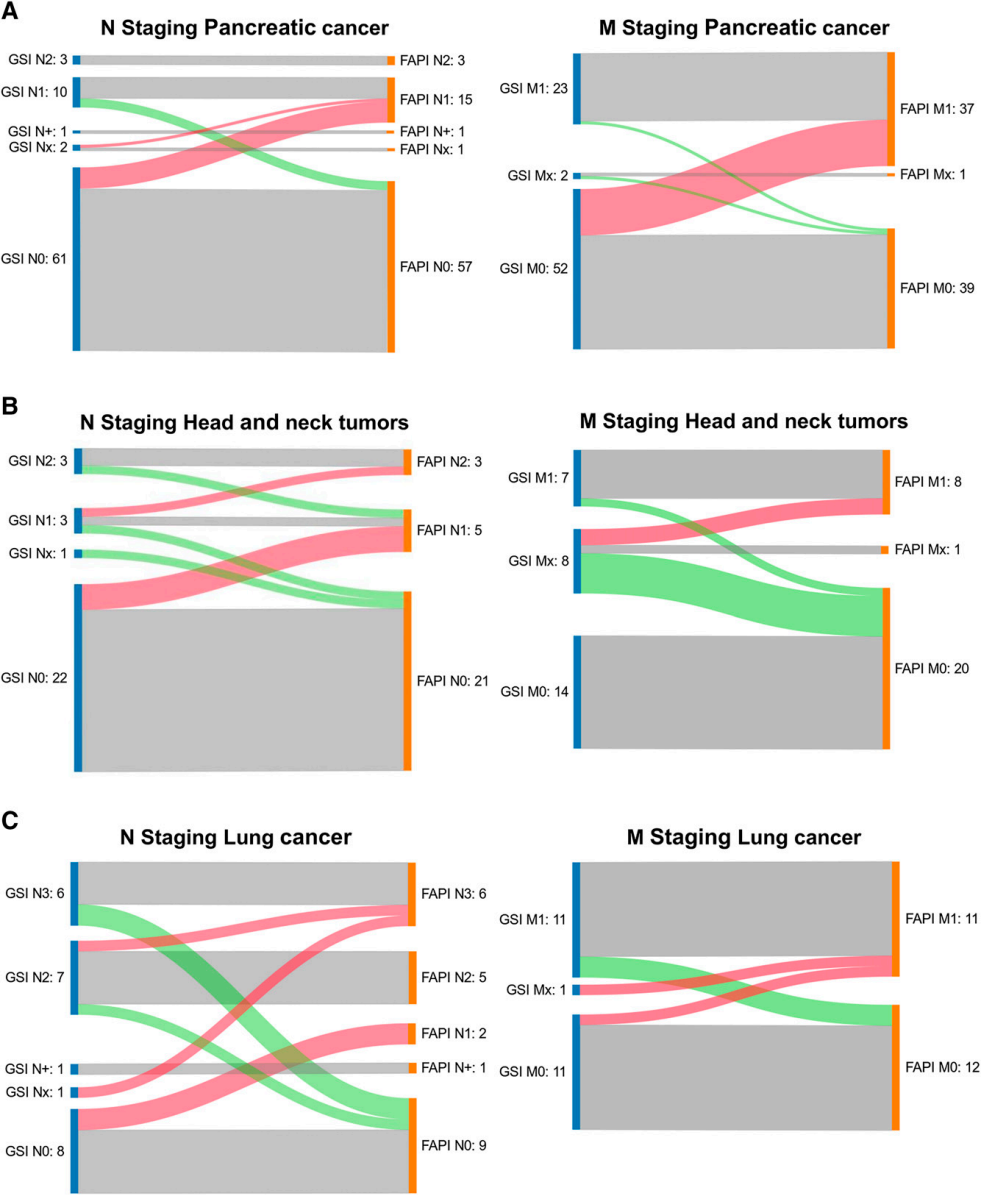
Sankey plots displaying ^68^Ga-FAPI PET/CT–related changes in N and M staging of pancreatic cancer (A), head and neck tumors (B), and lung cancer (C). Gray boxes indicate identical staging based on GSI and ^68^Ga-FAPI PET/CT. Red curves indicate upstaging based on ^68^Ga-FAPI PET/CT compared with GSI. Green curves indicate downstaging based on ^68^Ga-FAPI PET/CT compared with GSI.

Table 1 lists GSI modalities according to the types of cancer. Changes in TNM stage, numeric changes, and the location of metastases were recorded. Staging changes comparing ^68^Ga-FAPI PET with GSI were considered major according to the following criteria: T, any change in T stage or evidence of invasion of other organs by the primary tumor; N, a change from N0 to N+ or vice versa; and M, a change from M0 to M+ or detection of new metastases in other organs or vice versa. Minor staging changes were classified according to the following criteria: N, detection of additional lymph node metastases in N+-positive patients if not affecting N stage; and M, detection of additional distant metastases in the same organ or vice versa. Sankey plots for Figure 2 were produced using the freeware tool SankeyMATIC (www.sankeymatic.com).

**TABLE 1.** GSI Modalities According to Type of Cancer

Entity	Patients (*n*)	GSI
Pancreatic ductal adenocarcinoma	77	ceCT or ceMRI
Head and neck cancer	29	ceCT or ceMRI
Lung cancer	23	ceCT (supplementary ^18^F-FDG PET/CT for 5 patients)
Gliomas	21	ceMRI
Colorectal cancer	20	ceCT or ceMRI (supplementary ^18^F-FDG PET/CT for 5 patients)
Sarcomas	11	ceCT or ceMRI
Esophageal cancer	10	ceCT (supplementary ^18^F-FDG PET/CT for 1 patient)
Prostate cancer	3	ceCT and ceMRI (1) or ^18^F-PSMA PET/CT (2)
Thyroid cancer	5	ceCT or ceMRI (supplementary ^18^F-FDG PET/CT for 3 patients, supplementary ^68^Ga-DOTATOC PET/CT for 1 patient)
Ovarian cancer	4	^18^F-FDG PET/CT and supplementary ceCT or ceMRI
Breast cancer	2	ceCT (supplementary ^18^F-FDG PET/CT for 1 patient)
Hepatic cancer	4	ceCT or ceMRI
Cancer of unknown primary	1	ceCT
Melanoma	1	ceCT and ceMRI
Gastric cancer	1	ceCT
Cervical cancer	2	ceCT or ceMRI
Thymus cancer	1	ceCT
Lymphoma	1	ceCT
Gallbladder cancer	2	ceCT
Chordoma	1	ceCT
Renal cancer	1	ceCT
Esthesioneuroblastoma	1	ceCT
Cholangiocellular carcinoma	4	ceCT or ceMRI
Appendix cancer	1	ceCT
Total	226	

ceCT = contrast-enhanced CT; ceMRI = contrast-enhanced MRI.

### Evaluation of Impact of ^68^Ga-FAPI PET on Oncologic Management

Changes in clinical management related to additional findings on ^68^Ga-FAPI PET were recorded after retrospective review of patient charts by three of the authors. Changes in oncologic management (management based on ^68^Ga-FAPI PET/CT vs. management based on GSI) were classified as follows: fundamental alterations in the type or intent of treatment type were classified as major, whereas changes within an already prescribed treatment regime were classified as minor.

### Statistical Analysis

Data were analyzed descriptively by comparing numeric results and percentages of TNM changes and changes in oncologic management.

## RESULTS

### Patient Characteristics

Our cohort consisted of 137 male and 89 female patients with a mean age of 62 y (range, 20–86 y). In 48 patients imaging was performed at initial diagnosis, whereas in 50 patients imaging was performed for assessing metastatic disease. In 34 patients imaging was obtained for progressive disease, in 77 patients it was obtained for follow-up, and in 14 patients it was obtained in the adjuvant setting after surgery. In 3 cases the clinical situation could not be determined. The most common tumor site was pancreatic cancer (77 patients), head and neck tumors (29 patients), and lung cancer (23 patients). The cohort also included some rare tumors such as uterine sarcoma, appendiceal carcinoma, and thymus cancer. Oncologic diagnoses and clinical characteristics are listed by tumor type in Supplemental Table 1 (supplemental materials are available at http://jnm.snmjournals.org).

### Impact of ^68^Ga-FAPI PET on TNM Staging

Among 205 patients, 86 (42%) experienced a TNM change after ^68^Ga-FAPI PET/CT. For most (91%) of these cases, a major change was observed, and upstaging (53 major, 6 minor) was more frequent than downstaging (29 major, 2 minor). The most frequent reason for upstaging was the detection of new metastases on ^68^Ga-FAPI PET/CT compared with GSI. Three patients with carcinoma of the gallbladder (1) and lung cancer (2) had both major upstaging and major downstaging after ^68^Ga-FAPI PET/CT. In addition, 1 patient with lung cancer showed major downstaging and minor upstaging. Major changes occurred most frequently for pancreatic cancer patients (26 upstaged, 6 downstaged) and lung cancer (5 upstaged, 9 downstaged). Table 2 provides an overview of changes in TNM staging for all patients, and Supplemental Table 5 provides changes in TNM staging of rare entities. Figure 2 depicts changes in N and M staging of the 3 most common entities—pancreatic cancer, head and neck tumors, and lung cancer—in Sankey plots. Supplemental Tables 2–4 provide an overview of the locations of the additional findings on ^68^Ga-FAPI PET/CT compared with GSI, as well as ^68^Ga-FAPI–negative lesions, which led to staging changes in these 3 entities. For 21 patients with glioma, there were no TNM staging guidelines.

**TABLE 2.** Changes in TNM Staging

Entity	Patients (*n*)	Staging					Explanatory note
		Unchanged	Major up	Major down	Minor up	Minor down	
PDAC	77	44	26	6	1	0	
HNC	29	19	6	2	1	1	
Lung cancer	23	10	5	9	2	0	In 2 cases, major upstaging and major downstaging at same time; in 1 case, major downstaging and minor upstaging at same time
Gliomas	21						
Colorectal cancer	20	10	6	3	1		
Sarcomas	11	6	2	3			
Esophageal cancer	10	5	1	2	1	1	
Thyroid cancer	5	3	1	1			
Others*	30	22	6	3	0	0	In 1 case, major up- and downstaging at same time
Total number	226	119	53	29	6	2	

* Prostate cancer, ovarian cancer, breast cancer, hepatic cancer, cancer of unknown primary, melanoma, gastric cancer, cervical cancer, thymus cancer, lymphoma, gallbladder cancer, chordoma, renal cancer, esthesioneuroblastoma, cholangiocellular carcinoma, appendix cancer.

PDAC = pancreatic ductal adenocarcinoma; HNC = head and neck cancer.

### Impact of ^68^Ga-FAPI PET on Patient Management

Among the 226 patients who underwent ^68^Ga-FAPI PET/CT for staging or restaging, 18 had no further clinical information available. Of the remaining 208 patients, 117 (56.3%) had a change in clinical management; however, major changes in management occurred in only 14 patients (12%). The major changes in management after ^68^Ga-FAPI PET/CT included irradiation of a new organ, ^68^Ga-FAPI radioligand therapy, chemotherapy in place of radiation therapy, additional treatments such as surgery or chemotherapy, or a change in treatment intent (curative vs. palliative). Major treatment changes due to ^68^Ga-FAPI PET/CT more frequently led to systemic therapy in place of local treatment (4 cases) or local treatment in place of systemic treatment (1 case). Among the minor changes caused by findings on ^68^Ga-FAPI PET/CT, the most frequent was adjustment of the target volume for patients undergoing radiation therapy. Clinical management changed most frequently for pancreatic cancer (7 major, 23 minor), lung cancer (1 major, 13 minor), and head and neck tumors (1 major, 24 minor). Table 3 shows the changes in oncologic management for all cancer types, and Supplemental Table 6 shows changes in oncologic management for each of the rare entities.

**TABLE 3.** Changes in Oncologic Management

Entity	Patients (*n*)	Clinical management			
		Unchanged	Major	Minor	Unknown
PDAC	77	34	7	23	13
HNC	29	4	1	24	
Lung cancer	23	8	1	13	1
Gliomas	21	6		15	
CRC	20	9	3	7	1
Sarcomas	11	5	0	5	1
Esophageal cancer	10	2		8	
Thyroid cancer	5	4			1
Others*	30	19	2	8	1
Total number	226	91	14	103	18

* Prostate cancer, ovarian cancer, breast cancer, hepatic cancer, cancer of unknown primary, melanoma, gastric cancer, cervical cancer, thymus cancer, lymphoma, gallbladder cancer, chordoma, renal cancer, esthesioneuroblastoma, cholangiocellular carcinoma, appendix cancer.

PDAC = pancreatic ductal adenocarcinoma; HNC = head and neck cancer; CRC = colorectal cancer.

### Differences Between Clinical Settings

**FIGURE 3.**
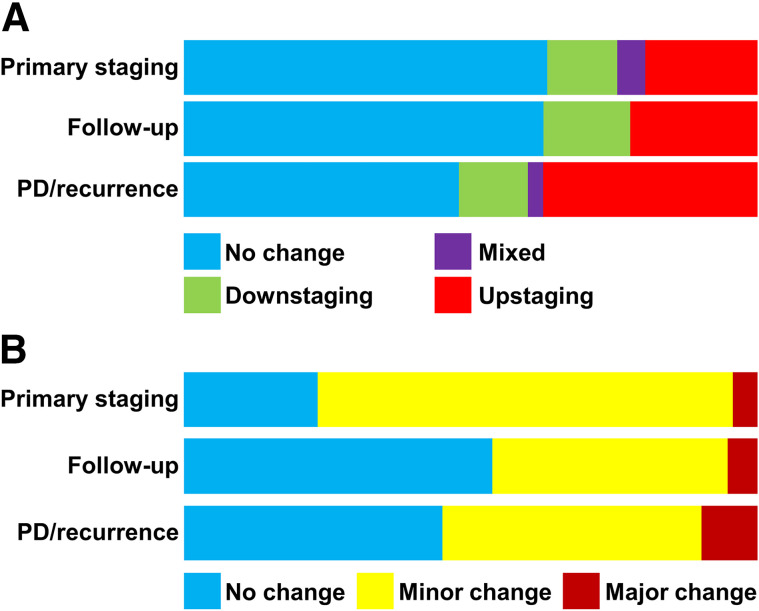
Relative distribution of ^68^Ga-FAPI PET–related changes in TNM staging (A) and clinical management (B) in different clinical settings (primary staging, follow-up, and progressive disease [PD]/recurrence). Bars are scales to 100% of patients analyzed per group.

To evaluate the potential influence of disease state on the impact of ^68^Ga-FAPI PET/CT, we divided our cohort into 3 subgroups: primary staging (*n* = 48), follow-up or adjuvant therapy (*n* = 91), and progressive disease or recurrence (*n* = 84). Three patients for whom the clinical setting remained unclear were excluded (Table 4). ^68^Ga-FAPI PET/CT led to changes in TNM staging less frequently in the primary and follow-up settings than in the progressive disease/recurrence setting. However, the impact on oncologic management was highest in the primary setting, followed by progressive disease/recurrence and finally follow-up, mostly because of ^68^Ga-FAPI PET/CT–related changes in radiotherapy planning (Fig. 3).

**TABLE 4.** Cohort Divided into 3 Subgroups: Primary Staging, Follow-up or Adjuvant Therapy, and Progressive Disease or Recurrence

Subgroup	Patients (*n*)	TNM staging					Oncologic management			
		No change	Downstaging	Mixed	Upstaging	Gliomas	No change	Minor change	Major change	No sufficient data
Primary staging	48	26	5	2	8	7	11	34	2	1
Follow-up	91	54	13	0	19	5	42	32	4	13
Progressive disease	84	36	9	2	28	9	37	37	8	2
Unclear	3	3	0	0	0	0	1	0	0	2
Total	226									

### Case Vignettes

#### Case 1

**FIGURE 4.**
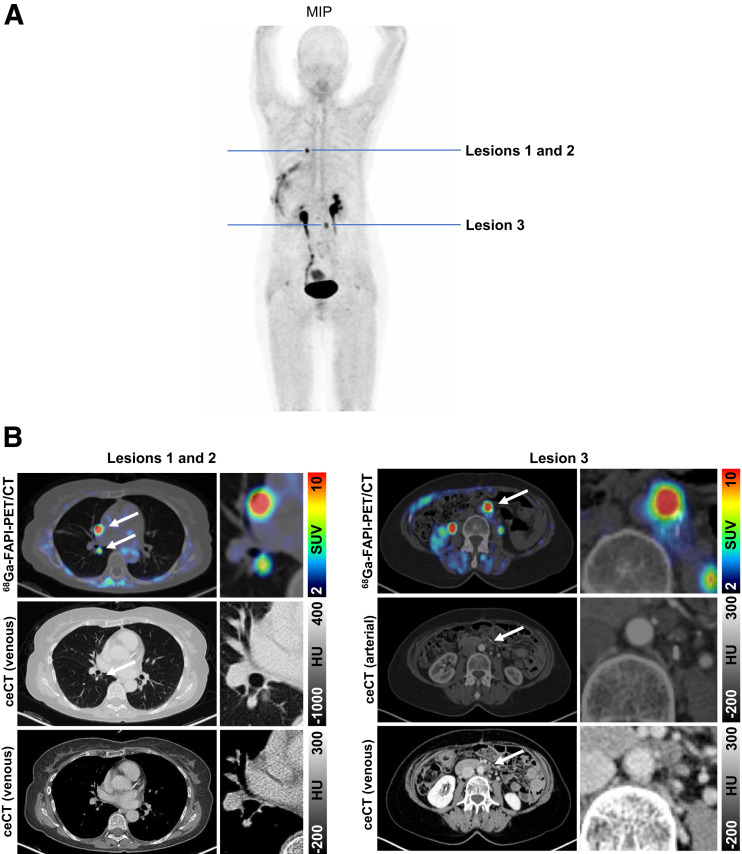
Example images of 64-y-old woman with recurrent pancreatic ductal adenocarcinoma. (A) Maximum-intensity projection (MIP) of ^68^Ga-FAPI PET. (B) Axial ^68^Ga-FAPI PET/CT images and contrast-enhanced CT (ceCT) images of suggestive lesions (arrows: lesions 1 and 2, pulmonary metastasis and mediastinal lymph node metastasis; lesion 3, paraaortic lymph node metastasis) detected by ^68^Ga-FAPI PET. HU = Hounsfield units.

A 64-y-old woman with pancreatic ductal adenocarcinoma achieved a complete remission after resection of the tail of the pancreas, with splenectomy, lymphadenectomy, and adjuvant chemotherapy (FOLFIRINOX [fluorouracil, leucovorin, irinotecan, and oxaliplatin] and oxaliplatin) and resection of the paraaortic lymph nodes and pulmonary metastases (Fig. 4). The tumor marker CA 19.9 had increased from 960 to 1,600 ng/mL, but restaging with contrast-enhanced CT did not reveal any sites of recurrence. A ^68^Ga-FAPI PET/CT scan revealed pulmonary metastases with mediastinal and paraaortic lymph node metastases. Retrospectively, we observed faint radiologic correlates for these metastases on previous CT scans, which were not prospectively interpreted as positive. Thus, ^68^Ga-FAPI PET/CT restaged the patient from cT0cN0cM0 to cTxcN1cM1, leading to a major change in oncologic management. Previously, the patient was being observed, but after the ^68^Ga-FAPI PET/CT scan, systemic chemotherapy (FOLFIRINOX followed by 5-fluorouracil) was administered and resulted in regression of the pulmonary and lymphatic tumor lesions.

#### Case 2

**FIGURE 5.**
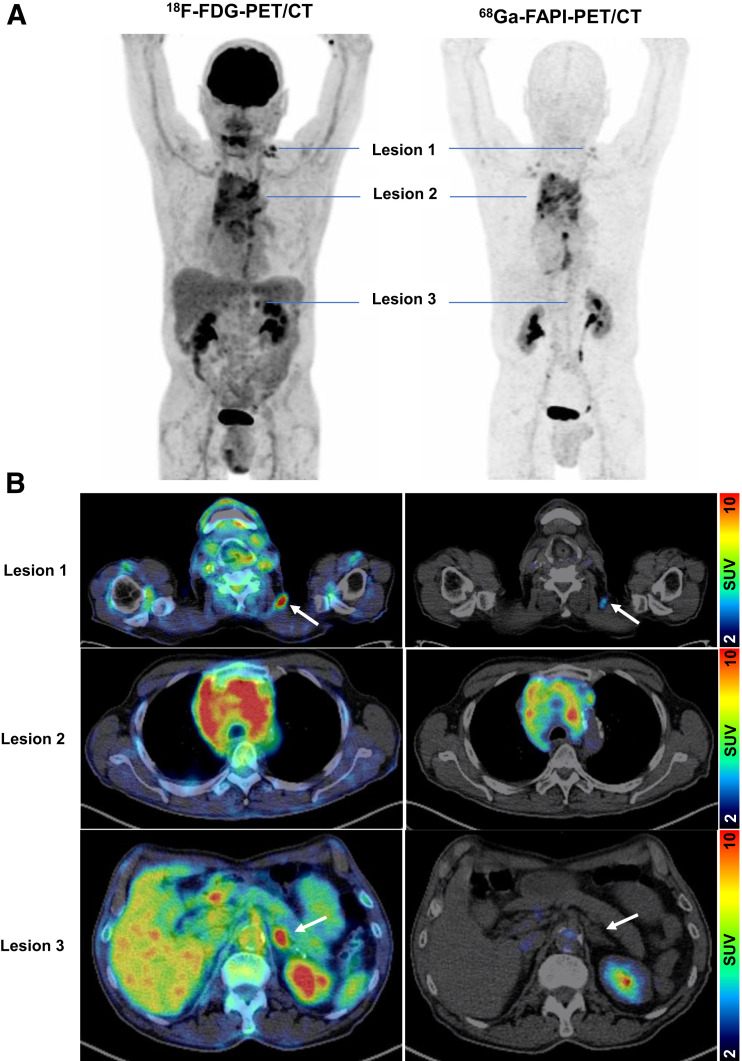
Example images of 78-y-old man with primary non–small cell lung cancer. (A) Maximum-intensity projection of ^18^F-FDG PET and ^68^Ga-FAPI PET. (B) Axial ^18^F-FDG PET/CT and ^68^Ga-FAPI PET/CT of suggestive lesions (arrows: lesion 1, cervical lymph node metastasis; lesion 2, non–small cell lung cancer with involvement of mediastinal lymph nodes; lesion 3, left adrenal mass). Although lesions 1 and 2 were clearly detectable by both PET scans, lesion 3 was ^18^F-FDG–avid but not confirmed by ^68^Ga-FAPI PET.

A 78-y-old man presented with primary non–small cell lung cancer. GSI with ^18^F-FDG PET/CT showed extensive mediastinal involvement, with cervical lymph node and adrenal metastases (Fig. 5). ^68^Ga-FAPI PET/CT confirmed the primary and cervical lymph node disease, but the adrenal mass did not demonstrate uptake and was therefore reassessed as more likely benign. Before the ^68^Ga-FAPI PET/CT, the patient was to undergo selective irradiation of the left adrenal gland, but this was cancelled after the scan. The patient underwent definitive irradiation of the mediastinum and cervical lymph nodes.

## DISCUSSION

Although the sensitivity for detecting cancers with ^68^Ga-FAPI PET/CT has been well reported, to date there is little information on the clinical impact of ^68^Ga-FAPI PET/CT on oncologic patients. Our large-cohort–based results substantiate smaller studies, in which the impact of ^68^Ga-FAPI PET on the staging and management of pancreatic ductal adenocarcinoma and on the staging of adenoid cystic carcinoma was reported (15*,*^16^). In the current analysis, we observed that ^68^Ga-FAPI PET/CT resulted in changes in TNM staging in about 40% of patients. The impact on TNM staging was particularly pronounced in clinical settings of progressive disease and recurrence, which is in line with our previous findings in pancreatic ductal adenocarcinoma staging (15). In this diverse group of cancers, ^68^Ga-FAPI PET/CT had the largest impact on pancreatic and lung cancer. Although ^18^F-FDG PET/CT is clinically well established in lung cancer, ^68^Ga-FAPI PET/CT appeared to surpass ^18^F-FDG PET/CT in detecting additional disease, thus altering TNM staging. For instance, whereas ^18^F-FDG PET/CT altered staging in 35% of patients with non–small cell lung cancer, compared with CT, in this study ^68^Ga-FAPI PET/CT altered staging in 56% of such patients. (25). Similar results were observed for ^18^F-FDG PET/CT for gastric adenocarcinoma, hepatic carcinoma, and pancreatic cancer (26–29). The PET-PANC trial demonstrated that ^18^F-FDG PET/CT correctly changed the staging of pancreatic cancer in only 56 of 550 patients. However, ^18^F-FDG PET/CT influenced management in 250 (45%) patients and stopped resection in 58 (20%) patients who were due to have surgery (30). In the current study, only a small percentage of patients had prior PET imaging (^18^F-FDG, ^68^Ga-DOTATOC, ^68^Ga-PSMA) as part of their standard workup, and therefore, no comparison between ^68^Ga-FAPI and other PET tracers can be made. However, a structured head-to-head comparison of staging based on ^68^Ga-FAPI PET/CT and ^18^F-FDG PET/CT would be great interest, since the latter modality is so frequently performed as a standard of care.

The findings on ^68^Ga-FAPI PET/CT influencing TNM staging also had a direct impact on clinical management. More than half our cohort had a change in management due to findings on ^68^Ga-FAPI PET/CT that differed from GSI. A similar change of 30%–62% in patient management was seen after the introduction of PSMA PET/CT in prostate cancer, thus demonstrating the power of targeted PET agents to alter patient care (31–34). Interestingly, the greatest impact was on radiation therapy planning—for both PSMA PET/CT and ^68^Ga-FAPI PET/CT—which underscores the additional value of PET imaging for radiotherapy planning. In our cohort, PET imaging was able to enhance target volume delineation, leading to reduced exposure of organs at risk and improved definition of the target volume. This finding is in line with findings from a large PET trial for patients with non–small cell lung cancer undergoing chemoradiation. Nestle et al. concluded that ^18^F-FDG PET–based planning was able to improve local control at no cost of added toxicity (4). Similarly, ^68^Ga-FAPI PET/CT also decreases off-target exposure and improves the target volume delineation, resulting in improved dosimetry for radiotherapy. As a side note, ^68^Ga-FAPI PET/CT appears to be superior to other modalities in detecting peritoneal carcinosis, which is often difficult to detect on imaging. Several prior studies have suggested that ^68^Ga-FAPI PET/CT has a higher sensitivity and specificity in the detection of peritoneal lesions in ovarian and colorectal cancer (35*,*^36^). Our data suggest that ^68^Ga-FAPI PET/CT is an extremely promising diagnostic approach for peritoneal disease and is more sensitive and specific than contrast-enhanced CT and contrast-enhanced MRI.

This study had several limitations. First, this retrospective trial was conducted at a single institution; however, the large size of the study counters, to some extent, potential patient selection biases. Most of the findings on ^68^Ga-FAPI PET/CT were not histologically verified, although false positives can occur. There was also no evidence that the prescribed changes in patient management resulted in improved patient outcomes. Instead of 1 unified type of ^68^Ga-FAPI PET/CT, 4 chemical variants were used, creating additional variables. However, most of the agents tested here appear to perform similarly, reducing the impact of this factor. Because the exclusion criteria allowed for a relatively long interval between GSI and ^68^Ga-FAPI PET/CT (<100 d), we cannot fully exclude the possibility that in some patients the differences in TNM staging might have been due to actual disease progression. Thus, the results of this study must be considered preliminary. Despite these limitations, these results provide a basis for prospective randomized trials that can provide level 1 evidence of the value of ^68^Ga-FAPI PET/CT.

## CONCLUSION

This study demonstrated that ^68^Ga-FAPI PET/CT impacts both TNM staging and oncologic management in a high percentage of cancer patients with a variety of cancer types. ^68^Ga-FAPI PET/CT detected numerous malignant lesions (in particular lung cancer, pancreatic cancer, and head and neck cancers) not visible on standard imaging and helped radiation therapy planning achieve superior target delineation. This innovative technology offers the potential to improve outcomes for patients by better defining the full extent of their disease.

## DISCLOSURE

Stefan Koerber reports research grants from Viewray Inc. and honoraria from IBA Dosimetry and Think Wired! (outside the submitted work). Frederik Giesel is an advisor at ABX, Telix, SOFIE Biosciences, and α-Fusion and holds shares in the consultancy group iTheranostic. Jakob Liermann is funded by the Physician-Scientist Program of Heidelberg University, Faculty of Medicine. Thomas Walle reports stock ownership for Roche, Bayer, and Innate Pharma and research funding (outside the submitted work) from CanVirex AG, Basel, Switzerland, and the Institute of Clinical Cancer Research IKF GmbH, Frankfurt, Germany. Dirk Jaeger reports consulting fees from CureVac AG, Definiens, F. Hoffmann-La Roche Ltd., Genmab A-S, Life Science Inkubator GmbH, VAXIMM AG, OncoOne Research & Development Research GmbH, Oncolytics Biotech Inc., Zelluna, HDIT GmbH, AYOXXA, Seattle Genetics, BreakBio Corp., and Roche Pharma AG; received honoraria from SKK Kliniken Heilbronn GmbH, Georg Thieme Verlag, Terrapinn, Touch Medical Media, BMS GmbH & Co. KGaA, MSD, Guppe 5 Filmproduktion GmbH, AstraZeneca GmbH, the Department of Radiation Medicine at the University of Kentucky, the Norwegian Cancer Society Oslo, Wilhlem-Sander Stiftung, Else-Kröner-Fesenius Stiftung, Schering Stiftung, and NordForsk; and received support for attending meetings or travel from Amgen Inc., Oryx GmbH, Roche Glycart AG, Parexel.com, IKTZ HD GmbH, and BMS. Juergen Debus received grants from Accuray International Sàrl, Merck Serono GmbH, CRI–the Clinical Research Institute GmbH, View Ray Inc., Accuray Inc., RaySearch Laboratories AB, Vision RT Limited, Astellas Pharma GmbH, Astra Zeneca GmbH, Solution Akademie GmbH, Ergomed PLC Surrey Research Park, Siemens Healthcare GmbH, Quintiles GmbH, NovoCure, Pharmaceutical Research Associates GmbH, Boehringer Ingelheim Pharma GmbH Co., PTW-Freiburg Dr. Pychlau GmbH, Nanobiotix A.A., and IntraOP Medical (outside the submitted work). Uwe Haberkorn has a patent application for quinolone‐based FAP‐targeting agents for imaging and therapy in nuclear medicine and has shares of the consultancy group iTheranostics (outside the submitted work). No other potential conflict of interest relevant to this article was reported.

## Notes

KEY POINTS **QUESTION:** Does the addition of ^68^Ga-FAPI PET/CT to GSI impact the TNM staging and clinical management of oncologic patients? **PERTINENT FINDINGS:** ^68^Ga-FAPI PET/CT altered TNM stage in 42% of all patients and resulted in changes in clinical management in 52% of all patients, underscoring its potential utility in the diagnostic workup of cancers. **IMPLICATIONS FOR PATIENT CARE:** ^68^Ga-FAPI PET/CT in addition to GSI impacts TNM staging and oncologic management in a high percentage of patients with various cancers, resulting in meaningful changes in treatment.
